# Supplementary material for: The Evolution and Characterization of the RNA Interference Pathways in Lophotrochozoa
Source: Genome Biol Evol. 2024 May 7;16(5):evae098. doi: 10.1093/gbe/evae098 (PMC11114477; doi:10.1093/gbe/evae098)
Supplement: evae098_Supplementary_Data [file evae098_supplementary_data.zip › Supplementary_materials.zip!/selected_miRNAs/NoGe_selected_miRNAs/mirdeep_pdf_files/NMRB01000426.1_Notospermus_geniculatus_strain_Ushimado_scaffold426,_whole_genome_shotgun_sequence_13052.pdf]

Provisional ID	:	NMRB01000426.1_Notospermus_geniculatus_strain_Ushimado_scaffold426,_whole_genome_shotgun_sequence_13052
Score total	:	13031.4
Score for star read(s)	:	3.9
Score for read counts	:	13023.1
Score for mfe	:	1.4
Score for randfold	:	0
Score for cons. seed	:	3
Total read count	:	25556
Mature read count	:	25540
Loop read count	:	0
Star read count	:	16

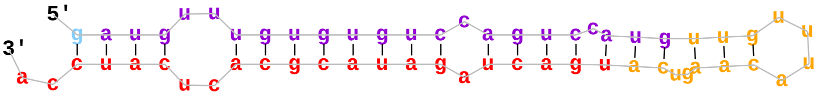

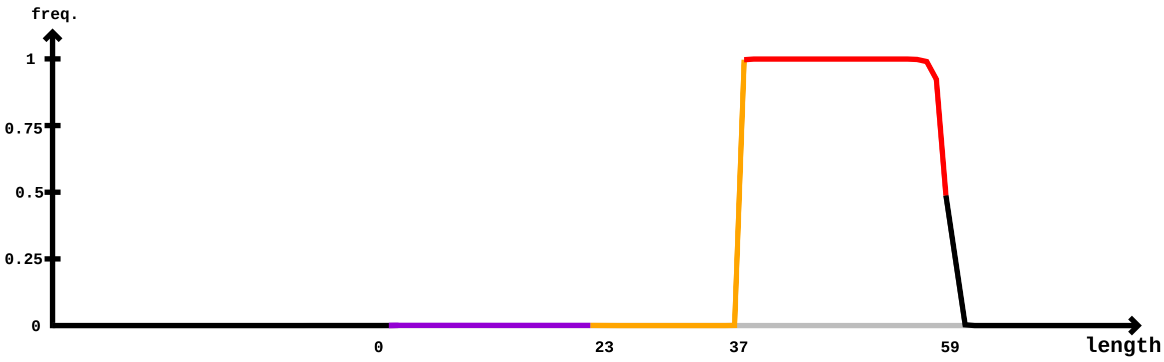

Star Mature

```
5'- gaacuuguugcccauuuguguuguguuuauguggauguuugugugucca guccauguuguuuacaagucaugacuagauacgcacucauccacucauacacagaaaccugc   -3'    obs
    gaacuuguugcccauuuguguuguguuuaugugggauguuugugugucca guccauguuguuuacaagucaugacuagauacgcacucauccacucauacacagaaaccugc          exp
    .................((.((((((....(((((((..((((((((.((((.((((((....)))..))))))).))))))))..)))))))....))))))..))....   reads   mm   sample
    .................................gauguuugugugucca guccaug.......................................................   3       0    Mm2
    ..................................auguuugugugucca guccaug.......................................................   3       0    Mm2
    ..................................auguuugugugucca guccaugu......................................................   1       0    Mm2
    ....................................................................caugacuagauacgcacucauc.....................   1       0    Mm2
    .....................................................................augacuagauacgcacucaucc....................   1       0    Mm2
    ......................................................................ugacuagauacgcacuca.......................   1       0    Mm2
    ......................................................................ugacuagauacgcacucau......................   4       0    Mm2
    ......................................................................ugacuagauGcgcacucauc.....................   1       1    Mm2
    ......................................................................ugGcuagauacgcacucauc.....................   1       1    Mm2
    ......................................................................ugacuagauacgcacucauU.....................   4       1    Mm2
    ......................................................................ugacuagauacgcacucauc.....................   103     0    Mm2
    ......................................................................ugacuagauaGgcacucauc.....................   1       1    Mm2
    ......................................................................ugacuagauacgcacucaucU....................   30      1    Mm2
    ......................................................................ugacuagauacgcacucCucc....................   4       1    Mm2
    ......................................................................ugacuGgauacgcacucaucc....................   25      1    Mm2
    ......................................................................ugacuagauacgcacucauAc....................   1       1    Mm2
    ......................................................................ugacuagauacgcacuAaucc....................   4       1    Mm2
    ......................................................................ugacuagauacgcacucaucc....................   766     0    Mm2
    ......................................................................ugacuagauacgcacuUaucc....................   2       1    Mm2
    ......................................................................ugacuagauacgGacucaucc....................   1       1    Mm2
    ......................................................................ugacuagauacgcacucGucc....................   2       1    Mm2
    ......................................................................uAacuagauacgcacucaucc....................   1       1    Mm2
    ......................................................................ugacuagauacAcacucaucc....................   2       1    Mm2
    ......................................................................ugacuagauaAgcacucaucc....................   2       1    Mm2
    ......................................................................ugacuaNauacgcacucaucc....................   1       1    Mm2
    ......................................................................ugacuagauacgcacuGaucc....................   1       1    Mm2
    ......................................................................ugacuagauacgcacucaucA....................   5       1    Mm2
    ......................................................................ugGcuagauacgcacucaucc....................   1       1    Mm2
    ......................................................................ugacuagauaGgcacucaucc....................   2       1    Mm2
    ......................................................................ugacuagauacgcacucUucc....................   1       1    Mm2
    ......................................................................ugacuagauaUgcacucaucc....................   1       1    Mm2
    ......................................................................ugacuagauCcgcacucaucc....................   1       1    Mm2
    ......................................................................ugacuagauacgcacuAaucca...................   2       1    Mm2
```

Star **Mature**

gaacuuguugcccauuuguguuguguuuauguggauguuugugugucc aguccauguuguuuuacaagucaugacuagauacgcacucauccacucauacacagaaaccugc			
....ugacuagauacgcacucaucca...................	232	0	Mm2
....Agacuagauacgcacucaucca...................	1	1	Mm2
....ugacuagauacgcacucauccG...................	23	1	Mm2
....ugacuagauacgcacuUaucca...................	1	1	Mm2
....ugCcuagauacgcacucaucca...................	1	1	Mm2
....uAacuagauacgcacucaucca...................	1	1	Mm2
....ugacuagauacgcacucUucca...................	1	1	Mm2
....ugacuagauacgcacucauccC...................	3	1	Mm2
....ugacuagauacgcacucauccU...................	310	1	Mm2
....ugacuGgauacgcacucaucca...................	4	1	Mm2
....ugacuagauacgcacucauccaA..................	1	1	Mm2
....ugacuagauacgcacucauccaU..................	11	1	Mm2
....ugacuagauacgcacucauccaAu.................	1	1	Mm2
....ugacuagauacgcacucauccaUu.................	2	1	Mm2
....gacuGgauacgcacucaucc....................	1	1	Mm2
....gacuagauacgcacucaucc....................	2	0	Mm2
....gacuagauacgcacucauccU...................	1	1	Mm2
....ugacuagauacgcacuca.......................	22	0	Mm1
....ugacuagauacgcacucau......................	17	0	Mm1
....ugacuagauCcgcacucau......................	1	1	Mm1
....uUacuagauacgcacucauc.....................	1	1	Mm1
....ugacuagauacgcacucGuc.....................	3	1	Mm1
....ugacuagauacgcacucauc.....................	342	0	Mm1
....ugacuagauacgcacuAauc.....................	2	1	Mm1
....ugacuagauaGgcacucauc.....................	1	1	Mm1
....ugUcuagauacgcacucauc.....................	1	1	Mm1
....ugacuagauacAcacucauc.....................	1	1	Mm1
....ugacuagauacgcacucCuc.....................	1	1	Mm1
....ugacuagauCcgcacucauc.....................	3	1	Mm1
....ugacuagauacgcacucUuc.....................	1	1	Mm1
....ugacCagauacgcacucauc.....................	1	1	Mm1
....ugacuagauacgcacuUauc.....................	2	1	Mm1
....ugacuagauacgcacucauU.....................	18	1	Mm1
....ugacuagauaAgcacucaucc....................	3	1	Mm1
....ugacuagauacgcacuAaucc....................	4	1	Mm1
....ugGcuagauacgcacucaucc....................	1	1	Mm1
....ugacuagauacAcacucaucc....................	2	1	Mm1
....ugacuagauaGgcacucaucc....................	3	1	Mm1
....ugacuagauacgcUcucaucc....................	3	1	Mm1
....ugacuaUauacgcacucaucc....................	1	1	Mm1
....ugaGuagauacgcacucaucc....................	1	1	Mm1
....ugacuagauacgcacucaucU....................	45	1	Mm1
....ugUcuagauacgcacucaucc....................	1	1	Mm1
....ugaUuagauacgcacucaucc....................	1	1	Mm1
....ugacuagauacgcacucGucc....................	9	1	Mm1
....ugacuNgauacgcacucaucc....................	2	1	Mm1
....ugacuagauacgUacucaucc....................	1	1	Mm1
....ugacuGgauacgcacucaucc....................	32	1	Mm1
....uCacuagauacgcacucaucc....................	1	1	Mm1
....Cgacuagauacgcacucaucc....................	1	1	Mm1
....ugacuagauaUgcacucaucc....................	2	1	Mm1
....ugacuagauacgcacucaucA....................	1	1	Mm1
....ugacuagauacgAacucaucc....................	1	1	Mm1
....ugacuagauacgcacucUucc....................	3	1	Mm1
....ugCcuagauacgcacucaucc....................	1	1	Mm1
....ugacuagauUcgcacucaucc....................	1	1	Mm1
....Agacuagauacgcacucaucc....................	1	1	Mm1
....ugaAuagauacgcacucaucc....................	1	1	Mm1
....uAacuagauacgcacucaucc....................	2	1	Mm1
....ugacuagauacgcacucaucc....................	1865	0	Mm1
....ugacuagauCcgcacucaucc....................	4	1	Mm1
....ugacuagauacgcacucauAc....................	1	1	Mm1
....ugacuagauacgcacucCucc....................	3	1	Mm1
....Ngacuagauacgcacucaucc....................	1	1	Mm1
....ugacuagauacgcacuUaucc....................	3	1	Mm1
....ugacuagauacgcacucauccU...................	501	1	Mm1
....ugacuagauacgcacuAaucca...................	1	1	Mm1
....Ngacuagauacgcacucaucca...................	1	1	Mm1
....ugacuagauacgcacuGaucca...................	1	1	Mm1

```
                                  Star                                Mature
gaacuuguugcccauuuguguuguguuuauguggauguuuguguguccaguccauguuguuuacaagucaugacuagauacgcacucauccacucauacacagaaaccugc
......................................................................ugacuagauacgcacucUucca...................   1   1   Mm1
......................................................................ugacuagauacgcacucauccG...................   3   1   Mm1
......................................................................ugacuagauacgcacucaucca...................   223 0   Mm1
......................................................................ugacuagauacgcacucauccC...................   7   1   Mm1
......................................................................ugacuagauacgcacucGucca...................   1   1   Mm1
......................................................................ugacuagauacgcacucauccaU..................   3   1   Mm1
......................................................................ugacuagauacgcacucauccaUu.................   16  1   Mm1
........................................................................acuagauacgcacucaucc.....................   1   0   Mm1

..................................auguuuguguguccaguccaug.......................................................   6   0   Fm2
..................................auguuuguguguccaguccaGg.......................................................   1   1   Fm2
......................................................................ugacuagauacgcacucau......................   2   0   Fm2
......................................................................ugacuagauCcgcacucau......................   1   1   Fm2
......................................................................ugacuagauacgcacuAauc.....................   1   1   Fm2
......................................................................ugacuagauacgUacucauc.....................   1   1   Fm2
......................................................................ugacuagauacgcacucauc.....................   143 0   Fm2
......................................................................ugacuGgauacgcacucauc.....................   4   1   Fm2
......................................................................ugacuagauaAgcacucauc.....................   1   1   Fm2
......................................................................Ngacuagauacgcacucauc.....................   1   1   Fm2
......................................................................ugacuagauacgcacuGaucc....................   1   1   Fm2
......................................................................ugacuagauacgcacucGucc....................   2   1   Fm2
......................................................................ugacuagauacgcacucaucc....................   555 0   Fm2
......................................................................ugacuagauacgcacuAaucc....................   1   1   Fm2
......................................................................ugacuagauacgcacucaucA....................   4   1   Fm2
......................................................................ugacuagauacgcacucUucc....................   2   1   Fm2
......................................................................ugacuagauaAgcacucaucc....................   2   1   Fm2
......................................................................ugacuagauacgcacucCucc....................   3   1   Fm2
......................................................................ugacGagauacgcacucaucc....................   1   1   Fm2
......................................................................ugacuagauacgcacucaucU....................   13  1   Fm2
......................................................................ugacCagauacgcacucaucc....................   1   1   Fm2
......................................................................ugacuagauacAcacucaucc....................   6   1   Fm2
......................................................................ugacuagauCcgcacucaucc....................   1   1   Fm2
......................................................................ugaAuagauacgcacucaucca...................   2   1   Fm2
......................................................................ugacuagauacgcacuAaucca...................   1   1   Fm2
......................................................................ugacuagaAacgcacucaucca...................   1   1   Fm2
......................................................................ugacuagauacgcacucauccU...................   258 1   Fm2
......................................................................ugacuagauacgcacucaucca...................   193 0   Fm2
......................................................................ugacuagauaGgcacucaucca...................   1   1   Fm2
......................................................................ugacuagauacgcacucauccG...................   19  1   Fm2
......................................................................ugacuagauacgcacuUaucca...................   1   1   Fm2
......................................................................ugacuagauacgcacucGucca...................   1   1   Fm2
......................................................................ugacuagauacgcacucauccaAu.................   1   1   Fm2
.......................................................................gacuagauacgcacucaucca...................   1   0   Fm2

.........................uuuauguggauguuugugu...................................................................   1   0   Fm1
......................................................................ugacuagauacgcacuAau......................   1   1   Fm1
......................................................................ugacuagauacgcacucau......................   4   0   Fm1
......................................................................ugacuaUauacgcacucauc.....................   1   1   Fm1
......................................................................ugacuagauacgcacuAauc.....................   1   1   Fm1
......................................................................ugacuagauacgcacuGauc.....................   2   1   Fm1
......................................................................ugacuagauacgcacucUuc.....................   1   1   Fm1
......................................................................ugacuagauacgcacucauA.....................   6   1   Fm1
......................................................................ugacuUgauacgcacucauc.....................   1   1   Fm1
......................................................................ugacuagauaUgcacucauc.....................   1   1   Fm1
......................................................................ugacuagauacgUacucauc.....................   1   1   Fm1
......................................................................ugacuagauacgcacucauU.....................   18  1   Fm1
......................................................................ugacuagauacgcacucauc.....................   238 0   Fm1
......................................................................ugacuagauacgcacuAaucc....................   5   1   Fm1
......................................................................Ngacuagauacgcacucaucc....................   1   1   Fm1
......................................................................ugacuagauacgcacuGaucc....................   1   1   Fm1
......................................................................ugacuagauCcgcacucaucc....................   1   1   Fm1
......................................................................ugacuagauacgcacucaucG....................   2   1   Fm1
......................................................................ugacuagauaUgcacucaucc....................   1   1   Fm1
......................................................................ugacuagauacgcacucGucc....................   4   1   Fm1
......................................................................ugacuagauacgcacucaucc....................   936 0   Fm1
......................................................................ugacuagauGcgcacucaucc....................   2   1   Fm1
......................................................................ugacuagaGacgcacucaucc....................   1   1   Fm1
......................................................................ugacuagauacgcacucauUc....................   1   1   Fm1
......................................................................ugacuagauacAcacucaucc....................   3   1   Fm1
......................................................................ugacuagauacgAacucaucc....................   1   1   Fm1
```

```
                                  Star                                Mature
gaacuuguugcccauuuguguuguguuuauguggauguuuguguguccaguccauguuguuuacaagucaugacuagauacgcacucaucacucauacacagaaaccugc
......................................................................ugacuGgauacgcacucaucc....................   7      1   Fm1
......................................................................ugacuagauacgcacuUaucc....................   4      1   Fm1
......................................................................ugGcuagauacgcacucaucc....................   2      1   Fm1
......................................................................ugacuagauacgcacucCucc....................   5      1   Fm1
......................................................................ugacuagaCacgcacucaucc....................   1      1   Fm1
......................................................................ugacuagauacgcGcucaucc....................   1      1   Fm1
......................................................................ugacuagauacgcacucaucU....................   46     1   Fm1
......................................................................ugacuagauacgcacucUucc....................   2      1   Fm1
......................................................................ugacuagauacgcacucauccU...................   307    1   Fm1
......................................................................ugaAuagauacgcacucaucca...................   1      1   Fm1
......................................................................ugacuagauaAgcacucaucca...................   1      1   Fm1
......................................................................ugacuagUuacgcacucaucca...................   1      1   Fm1
......................................................................ugacuagauaGgcacucaucca...................   1      1   Fm1
......................................................................ugacuagauacgcGcucaucca...................   1      1   Fm1
......................................................................ugacuagauacgcacucUucca...................   1      1   Fm1
......................................................................ugacuagauacgcacucaucca...................   195    0   Fm1
......................................................................ugacuagauacgcacucauccG...................   26     1   Fm1
......................................................................ugacuagauacgcacucaucAa...................   2      1   Fm1
......................................................................ugacuagauacgcacucauccaA..................   4      1   Fm1
......................................................................ugacuagauacgcacucauccaU..................   13     1   Fm1
......................................................................ugacuagauacgcacucauccaUu.................   3      1   Fm1
.......................................................................gacuagauacgcacucaucc....................   4      0   Fm1
.......................................................................gacuGgauacgcacucaucc....................   1      1   Fm1
.......................................................................gacuagauacgcacucaucca...................   3      0   Fm1

.............auuuguguuguguuuaugug..............................................................................   1      0   Mm3
.................................gauguuuguguguccaguccau........................................................   1      0   Mm3
.....................................................................Cugacuagauacgcacucaucc....................   1      1   Mm3
.....................................................................augacuagauacgcacucaucc....................   1      0   Mm3
.....................................................................Cugacuagauacgcacucaucca...................   1      1   Mm3
......................................................................ugacuagauacgcacuca.......................   2      0   Mm3
......................................................................ugacuagauacgcacucU.......................   1      1   Mm3
......................................................................ugacuagauacgcacucau......................   6      0   Mm3
......................................................................ugacuagauacgcacucGuc.....................   2      1   Mm3
......................................................................ugacuGgauacgcacucauc.....................   1      1   Mm3
......................................................................ugacuagauacgcacuAauc.....................   1      1   Mm3
......................................................................ugacuagauacgcacucauc.....................   105    0   Mm3
......................................................................ugacuagauacgcacucauU.....................   6      1   Mm3
......................................................................ugacuagauacgcacuGauc.....................   1      1   Mm3
......................................................................ugacuagauacAcacucauc.....................   1      1   Mm3
......................................................................ugacuagauacgUacucauc.....................   1      1   Mm3
......................................................................ugacuagauacgcacuUauc.....................   1      1   Mm3
......................................................................ugacuagauacgcacucUucc....................   1      1   Mm3
......................................................................ugacuagauacgcacuAaucc....................   1      1   Mm3
......................................................................ugacuagauacgcacucaucA....................   3      1   Mm3
......................................................................ugacuagauacgcacucaucc....................   959    0   Mm3
......................................................................ugacGagauacgcacucaucc....................   1      1   Mm3
......................................................................ugacuagauacgcacucaucU....................   33     1   Mm3
......................................................................ugacuagauacAcacucaucc....................   1      1   Mm3
......................................................................ugacuGgauacgcacucaucc....................   22     1   Mm3
......................................................................ugacuagGuacgcacucaucc....................   1      1   Mm3
......................................................................ugacuagauaGgcacucaucc....................   1      1   Mm3
......................................................................ugacuagauacgcacucGucc....................   3      1   Mm3
......................................................................ugacuagauacgcacucCucc....................   2      1   Mm3
......................................................................ugacuagauacgcUcucaucc....................   1      1   Mm3
......................................................................ugacuagauacgcacucaucG....................   1      1   Mm3
......................................................................ugacuagauaAgcacucaucc....................   4      1   Mm3
......................................................................ugacuagauaUgcacucaucc....................   2      1   Mm3
......................................................................ugacuagauCcgcacucaucc....................   8      1   Mm3
......................................................................ugacuagauacgcacucauccG...................   28     1   Mm3
......................................................................ugacuagauacgcacucauccC...................   5      1   Mm3
......................................................................ugacuagauacgcacucGucca...................   3      1   Mm3
......................................................................ugacuagauacgcacucaucca...................   388    0   Mm3
......................................................................ugacuagauacUcacucaucca...................   1      1   Mm3
......................................................................ugacuGgauacgcacucaucca...................   1      1   Mm3
......................................................................ugacuagauacgcacucCucca...................   1      1   Mm3
......................................................................ugacuagauacgcacuGaucca...................   3      1   Mm3
......................................................................Ngacuagauacgcacucaucca...................   1      1   Mm3
......................................................................ugacuagauacgcacucauccU...................   1155   1   Mm3
......................................................................ugacuagauaUgcacucaucca...................   1      1   Mm3
```

```
                                  Star                                Mature
gaacuuguugcccauuuguguuguguuuauguggauguuuguguguccaguccauguuguuuacaagucaugacuagauacgcacucauccacucauacacagaaaccugc
......................................................................ugacuagauacgcacuAaucca...................   2     1     Mm3
......................................................................ugacuagauacgcacuUaucca...................   1     1     Mm3
......................................................................ugacuagauacgcacucUucca...................   1     1     Mm3
......................................................................Agacuagauacgcacucaucca...................   2     1     Mm3
......................................................................ugacuagauacgcacucauccaG..................   1     1     Mm3
......................................................................ugacuagauacgcacucauccaA..................   11    1     Mm3
......................................................................ugacuagauacgcacucauccaU..................   18    1     Mm3
......................................................................ugacuagauacgcacucauccaAu.................   1     1     Mm3
......................................................................ugacuagauacgcacucauccaUu.................   9     1     Mm3
.......................................................................gacuagauCcgcacucaucc....................   1     1     Mm3
.......................................................................gacuagauacgcacucaucc....................   1     0     Mm3
.......................................................................gacuagauacAcacucaucc....................   1     1     Mm3
.......................................................................gacuagauacgcacucauccU...................   6     1     Mm3

..................................auguuuguguguccaguccaug.......................................................   1     0     Fm3
.....................................................................Cugacuagauacgcacucauc.....................   3     1     Fm3
.....................................................................augacuagauacgcacucaucc....................   2     0     Fm3
.....................................................................Cugacuagauacgcacucaucc....................   5     1     Fm3
......................................................................ugacuagauCcgcacuca.......................   1     1     Fm3
......................................................................ugacuagauacgcacucC.......................   1     1     Fm3
......................................................................ugacuagauacgcacucU.......................   3     1     Fm3
......................................................................ugacuagauacgcacuca.......................   7     0     Fm3
......................................................................ugacuagauacAcacuca.......................   1     1     Fm3
......................................................................ugacuagauacgcacucUu......................   1     1     Fm3
......................................................................ugacuagauaUgcacucau......................   1     1     Fm3
......................................................................ugacuagauacgcacucCu......................   1     1     Fm3
......................................................................ugacuagauacAcacucau......................   3     1     Fm3
......................................................................ugacuagauacgcacucGu......................   1     1     Fm3
......................................................................ugacuagauCcgcacucau......................   12    1     Fm3
......................................................................ugacuagauacgcacucau......................   144   0     Fm3
......................................................................ugacuagauacAcacucauc.....................   1     1     Fm3
......................................................................ugacuagauacgcacucUuc.....................   1     1     Fm3
......................................................................ugacuagauacgcacucauc.....................   625   0     Fm3
......................................................................ugacuagauacgcacucGuc.....................   2     1     Fm3
......................................................................ugaGuagauacgcacucauc.....................   1     1     Fm3
......................................................................ugacuagauaAgcacucauc.....................   2     1     Fm3
......................................................................ugacuagauCcgcacucauc.....................   2     1     Fm3
......................................................................ugacuagauacgcacuGauc.....................   2     1     Fm3
......................................................................ugacuagauacgcacuAauc.....................   1     1     Fm3
......................................................................ugacuagauacgcacuUauc.....................   1     1     Fm3
......................................................................ugacuagauacgcacucauU.....................   18    1     Fm3
......................................................................ugacuagauacgUacucauc.....................   6     1     Fm3
......................................................................ugacuagauUcgcacucaucc....................   1     1     Fm3
......................................................................ugacuagauacgcaUucaucc....................   2     1     Fm3
......................................................................ugacuagauacgcacuCUucc....................   10    1     Fm3
......................................................................ugacuaAauacgcacucaucc....................   1     1     Fm3
......................................................................ugacuagauacgcacucCucc....................   17    1     Fm3
......................................................................ugacuUgauacgcacucaucc....................   3     1     Fm3
......................................................................ugacuagauacgcacuGaucc....................   7     1     Fm3
......................................................................ugCcuagauacgcacucaucc....................   2     1     Fm3
......................................................................ugacuagauacgcacuUaucc....................   10    1     Fm3
......................................................................ugacuagauaUgcacucaucc....................   11    1     Fm3
......................................................................ugacuagauaGgcacucaucc....................   3     1     Fm3
......................................................................ugacuagauacgcacuAaucc....................   23    1     Fm3
......................................................................ugUcuagauacgcacucaucc....................   1     1     Fm3
......................................................................ugacuagauacgcacucaAcc....................   2     1     Fm3
......................................................................ugacuagauaAgcacucaucc....................   12    1     Fm3
......................................................................ugacuagauGcgcacucaucc....................   1     1     Fm3
......................................................................ugacuagauacgcacucaucA....................   17    1     Fm3
......................................................................ugacuagauCcgcacucaucc....................   19    1     Fm3
......................................................................ugacuagauacgcacucGucc....................   18    1     Fm3
......................................................................ugacuagauacAcacucaucc....................   24    1     Fm3
......................................................................ugacuagauacgAacucaucc....................   1     1     Fm3
......................................................................ugacuGgauacgcacucaucc....................   41    1     Fm3
......................................................................uAacuagauacgcacucaucc....................   3     1     Fm3
......................................................................ugGcuagauacgcacucaucc....................   1     1     Fm3
......................................................................ugacuagauacgUacucaucc....................   2     1     Fm3
......................................................................ugacuagauacgcacucaucc....................   5080  0     Fm3
......................................................................ugacuagauacgcacucauUc....................   2     1     Fm3
......................................................................ugacuagauacgcUcucaucc....................   2     1     Fm3
```

Star **Mature**

```
gaacuuguugcccauuuguguuguguuuauguggauguuuguguguccaguccauguuguuuacaagucaugacuagauacgcacucaucccucauacacagaaaccugc
......................................................................ugacuaNauacgcacucaucc....................  1     1  Fm3
......................................................................uUacuagauacgcacucaucc....................  3     1  Fm3
......................................................................ugacuagauacUcacucaucc....................  1     1  Fm3
......................................................................ugacuagauacgcacucaucU....................  270   1  Fm3
......................................................................ugaUuagauacgcacucaucc....................  1     1  Fm3
......................................................................ugacuagGuacgcacucaucc....................  1     1  Fm3
......................................................................ugaAuagauacgcacucaucc....................  2     1  Fm3
......................................................................ugacuagauacgcacCcaucc....................  1     1  Fm3
......................................................................ugacuagaAacgcacucaucc....................  1     1  Fm3
......................................................................ugacuaUauacgcacucaucc....................  2     1  Fm3
......................................................................Agacuagauacgcacucaucc....................  1     1  Fm3
......................................................................ugacuagUuacgcacucaucc....................  1     1  Fm3
......................................................................ugacuagauaGgcacucaucca...................  1     1  Fm3
......................................................................ugacuagauacgcacucGucca...................  5     1  Fm3
......................................................................ugacuagauacgcacucCucca...................  4     1  Fm3
......................................................................ugacuNgauacgcacucaucca...................  1     1  Fm3
......................................................................ugCcuagauacgcacucaucca...................  1     1  Fm3
......................................................................ugacuGgauacgcacucaucca...................  3     1  Fm3
......................................................................ugacuagauaAgcacucaucca...................  2     1  Fm3
......................................................................ugacuagauacgcacucUucca...................  6     1  Fm3
......................................................................ugacuagauacgcacucauccC...................  2     1  Fm3
......................................................................ugacuagauacgUacucaucca...................  2     1  Fm3
......................................................................ugacuagauacgcacucauccU...................  6809  1  Fm3
......................................................................ugacuagauacgcacuUaucca...................  4     1  Fm3
......................................................................ugaUuagauacgcacucaucca...................  1     1  Fm3
......................................................................ugGcuagauacgcacucaucca...................  2     1  Fm3
......................................................................ugacuagauacgcacuAaucca...................  7     1  Fm3
......................................................................ugacuagauacgcacucaucAa...................  14    1  Fm3
......................................................................ugacuagauacgcacuGaucca...................  1     1  Fm3
......................................................................ugacuagauaUgcacucaucca...................  5     1  Fm3
......................................................................ugacuagauacgcacucaucca...................  1348  0  Fm3
......................................................................ugacuagauacgcacucauccaA..................  3     1  Fm3
......................................................................ugacuagauacgcacucauccaU..................  129   1  Fm3
......................................................................ugacuagauacgcacucauccaG..................  6     1  Fm3
......................................................................ugacuagauacgcacucauccaAu.................  16    1  Fm3
......................................................................ugacuagauacgcacucauccaUu.................  26    1  Fm3
.......................................................................gacuagauacgcacucau......................  1     0  Fm3
.......................................................................gacuagauacgcacucaucc....................  3     0  Fm3
.......................................................................gacuagauacgcacucauccU...................  36    1  Fm3
```
